# The Effect of Liquids Activated by Plasma Generated with a Microwave Plasmatron and High-Frequency Glow Discharge on Cotton Plant Development

**DOI:** 10.3390/plants14030304

**Published:** 2025-01-21

**Authors:** Sergey A. Shumeyko, Denis V. Yanykin, Mark O. Paskhin, Vladimir I. Lukanin, Dmitry A. Zakharov, Maxim E. Astashev, Roman Y. Pishchalnikov, Ruslan M. Sarimov, Mukhsindjan Kh. Ashurov, Erkindjan M. Ashurov, Dilbar K. Rashidova, Muzaffar M. Yakubov, Aleksei M. Davydov, Victoriya V. Gudkova, Yuri K. Danileyko, Alexey S. Dorokhov, Sergey V. Gudkov

**Affiliations:** 1 Prokhorov General Physics Institute of the Russian Academy of Sciences, Vavilov Str. 38, 119991 Moscow, Russia; pashin.mark@mail.ru (M.O.P.); vladimirlukanin@yandex.ru (V.I.L.); zaharov121221@mail.ru (D.A.Z.); astashev@yandex.ru (M.E.A.); rpishchal@kapella.gpi.ru (R.Y.P.); rusa@kapella.gpi.ru (R.M.S.); freaman@mail.ru (A.M.D.); gudkova-vi@fpl.gpi.ru (V.V.G.); dyuk42@list.ru (Y.K.D.); s_makariy@rambler.ru (S.V.G.); 2 Institute of Basic Biological Problems of the Russian Academy of Sciences, Federal Research Center “Pushchino Scientific Center for Biological Research” FRC PSCBR of the Russian Academy of Sciences, Institutskaya Str. 2, 142290 Pushchino, Russia; 3 Institute of Nuclear Physics Academy of Sciences of the Republic of Uzbekistan, Tashkent 100214, Uzbekistan; ashurov49@mail.ru; 4 Limited Liability Company “Souvenir”, Tashkent 100117, Uzbekistan; erkin@souvenir-uz.com; 5 Cotton Breeding, Seed Production and Agritechnologies Research Institute Uzbekistan, Tashkent 111218, Uzbekistan; etoile111@yandex.com (D.K.R.); yakubov.m.m@mail.ru (M.M.Y.); 6 Federal Scientific Agroengineering Center VIM, 109428 Moscow, Russia; dorokhov.vim@yandex.ru; 7 Department of Biophysics, Lobachevsky State University, 23 Gagarin Avenue, 603950 Nizhny Novgorod, Russia

**Keywords:** plasma-activated water, plasma discharge, plasmotron, cotton plant, KNO_3_, plant growth, seed germination, water deficit, drought

## Abstract

In this study, we investigated the effect of plasma-activated liquids (PAL) on the cotton plant (*Gossypium hirsutum* L.) growth under laboratory and field conditions. We used two types of PAL: deionized water activated with plasma generated using a microwave plasmatron in atmospheric-pressure air flow (PAW) and a 1.5% KNO_3_ solution activated using plasma generated in an electrochemical cell (PAKNO_3_). These treatments differ in terms of their content of long-lived biologically active compounds. PAW contains a higher concentration of hydrogen peroxide (150 μM compared to 1.1 μM), while PAKNO_3_ is more saturated with NO_2_^−^ and NO_3_^−^ (1510 μM compared to 300 µM). We found that PAW improved cotton plant growth under field conditions and in a laboratory drought stress. Additionally, PAW increased field emergence and germination of heat-treated cotton seeds in the laboratory. It was revealed that PAW prevents the drought-induced disruption of the partitioning of absorbed light energy in the photosynthetic apparatus. Meanwhile, PAKNO_3_ has a positive effect on seed germination. The positive effect of PALs on cotton seeds and plants is thought to be due to the generation of long-lived biologically active oxygen and nitrogen species during plasma treatment of the liquid.

## 1. Introduction

Global population growth stimulates the demand for agricultural products [1]. Currently, traditional methods for increasing and securing the yield of cultivated crops are actively applied, which include, on the one hand, the use of chemical fertilizers and pesticides [2], which are potentially dangerous to the environment, as well as the cultivation of genetically modified plants, which are prohibited in many countries worldwide [3], and, on the other hand, the construction of greenhouses and hothouses, the application of hydroponic and aeroponic techniques, and drip irrigation [4]. However, given the limited availability of agricultural land, modern technologies for intensification agriculture are unable to meet the growing demand. To overcome this gap, new approaches for increasing crop productivity are being developed. Notable among these are the treatment of plants and/or their parts with a constant or alternating magnetic field [5], the application of photoconversion coatings in greenhouses and hothouses [6], the application of bacteria and biological stimulants, pretreatment of seeds with a plasma discharge [7], and the use of liquids treated with plasma (the so-called plasma-activated solutions, PAL) [8]. Such approaches may also be relevant for regions where agricultural production is sufficient. The chief aim in these regions is not maximum production but environmentally friendly and sustainable production.

Treatment of pure water or other liquid by various forms of plasma discharge leads to launch of cascade of redox reactions that results in changing of liquid`s chemical composition [9]. The main “active” components of PAL are reactive oxygen species (ROS) and reactive nitrogen species (RNS) [10]. Typically, most ROS and RNS generated during liquid activation are short-lived compounds; in the stable state, PAL contains only long-lived forms such as hydrogen peroxide, nitrate anion, nitrite anion, etc. The qualitative and quantitative composition of the resulting solutions depends on several factors: the activation method, the activation time, the working atmosphere, the structure of the plasma discharge, type of liquid, etc. [8]. There are two main activation methods: above the liquid [11] and in the liquid [12]. The most commonly used working gases are ambient air, molecular oxygen (O_2_), argon (Ar), helium (He), molecular nitrogen (N_2_) and their mixtures in various combinations [13]. Dielectric barrier discharge, plasma jet, glow discharge, spark discharge, corona discharge and sliding arc discharge are commonly used for PAL production [8]. PAL has long been successfully used in medicine and the food industry for the disinfection of equipment and products (scalpels, knives, cutting boards, etc.). Currently, an increasing number of studies are focused on the use of PAL in agriculture [10,14]. This is due to the fact that reactive oxygen and nitrogen species, which are produced as a result of plasma treatment solutions, have a significant influence on plants at both the cellular and organismal levels. ROS and RNS are known to be important signaling molecules in plants. These species improve seed germination, influence plant development [15], and modulate the effects of abiotic and biotic stress factors by enhancing protective mechanisms [16]. There are three main mechanisms of action of PAL on plant seeds. The first, ROS causes cracking and chemical alteration of the seed surface and accompanied changes in hydrophilicity of the outer seed coat, which leads to better absorption of water and nutrients [17,18]; the second, RNS can be used by the seed as a nutrient, since absorbed NO_3_^−^ is reduced by nitrate reductase to NO_2_, then by nitrite reductase to ammonium [19]; and the third, hydrogen peroxide and NO_3_^−^ suppress the synthesis of abscisic acid, which regulates seed germination and plant growth [15]. PALs can influence at the adult plant level because they contain an additional nitrogen source that can be used by plants [20], hydrogen peroxide, which, as a signaling molecule, can trigger a cascade of reactions, which reduce oxidative stress [21], and other ROS species, which affect the hormonal profile of plants and the expression of certain genes [22].

Despite significant progress in understanding the effects of PALs on crop plants, several issues remain poorly understood. For example, it is still unclear how PALs affect adult plants and their resistance to stress factors [8].

In this study, we investigated the effect of plasma-activated liquids (PAL) on the development of cotton plants (*Gossypium hirsutum* L.) throughout their ontogenesis from seed germination to harvest.

## 2. Results

To carry out the study, two types of PAL were produced. The basis for the first was deionized water activated in plasma generated by a microwave plasmatron at atmospheric pressure with an air flow (PAW) [23], and for the second a KNO_3_ solution (1.5%) activated using plasma generated in an electrochemical cell (PAKNO_3_) (for details, see Section 4). PAW had the following physicochemical properties: concentration of NO_2_^−^, NO_3_^−^ and H_2_O_2_ were 200 ± 20 μM, 100 ± 10 μM and 150 ± 20 μM, respectively, conductivity was 0.06 ± 0.002 S cm^−1^ and pH 5.6 ± 0.2. After activation in the electrochemical cell, PAKNO_3_ had the following characteristics: PAKNO_3_ have the following physicochemical properties: concentration of NO_2_^−^, NO_3_^−^ and H_2_O_2_ were 1320 ± 95 μM, 190 ± 28 μM and 1.1 ± 0.4 μM, respectively, conductivity was 0.035 ± 0.002 S cm^−1^ and pH 10.8 ± 0.2.

### 2.1. Effect of PAL on the Germination and Emergence of Cotton Seeds

The effect of PALs on cotton germination was studied under both laboratory and field conditions. It was shown that untreated cotton seeds exhibited 100% germination with 100% germination energy in laboratory conditions. Soaking seeds in all types of PAL did not lead to deterioration in germination parameters, which may indicate the absence of a negative effect of PALs on seeds (Table 1). In the field, the emergence of untreated seeds was lower (78.2%) than in the laboratory. Treatment of cotton seeds with PALs led to an increase in field germination to 84–86% in the case of using PAW_0.5%_ and PAW_0.75%_ and had not affected the seed germination in the case of using PAW_1%_. The decrease in cotton field emergence under field conditions may be due to the effect of unfavorable environmental factors, so we repeated the laboratory experiment using cotton seeds treated at 45 °C and 99% relative air humidity for 75 h. Heat treatment resulted in a decrease in seed germination (to 83.3%, which was comparable to the data obtained under field conditions). Germination of cotton seeds in the presence of PAL had a positive effect on seed germination: about 87–90% for PAW_0.5%_ seeds, PAW_0.75%_ seeds, PAKNO_3_ seeds, and KNO_3_ seeds. And only for PAW_1%_ seeds, the germination did not differ from the control seeds. Thus, an improvement in the quality of cotton seeds under the influence of PAL was demonstrated.

### 2.2. Effect of PAL Treatment of Cotton Seeds on Plant Growth and Development

**Figure 1:**
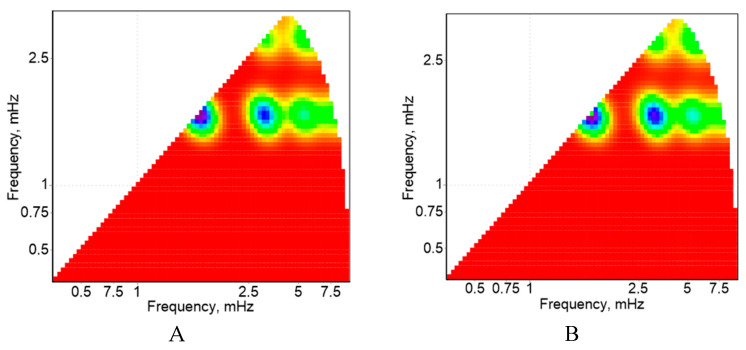
Values maps of fluctuations bispectrality coefficients in the background fluorescence level of not treated (**A**) and treated plants with plasma-activated water (**B**).

It was shown that cotton plants grown in the laboratory from seeds pre-soaked in deionized water or PALs exhibited similar stem and root growth (Table 2). Wavelet analysis has recently been shown to detect the effects of stress factors on plants at a time when visual changes are not yet observed [24]. We performed such a comparison of control plants and PAW_0.75%_ plants. The result of the complex continuous wavelet transform of the cotton plant fluorescence records under standard conditions and the result of the complex continuous wavelet transform of the plant fluorescence records treated with plasma-activated water did not differ significantly, although they contain distinct lines corresponding to the 10-min period of saturating light flashes, as well as doubled (second harmonic) and tripled (third harmonic) frequencies. The presence of harmonics indirectly indicates the nonlinearity of the reaction process to saturating flashes, since the flashes themselves are rectangular pulses. Often, the nonlinearity of the reaction process can be distinguished using maps of bispectrality coefficient values. The map of bispectrality coefficient values of the cotton plant fluorescence records under standard conditions is shown in Figure 1A, and the map of bispectrality coefficient values of the cotton plant fluorescence records treated with surfactants is shown in Figure 1B. It was also shown that there are also no significant differences between the maps of bispectrality coefficient values. Bispectrality analysis revealed only a high-frequency region (in the region of 1.8 MHz (millihertz), which corresponds to a period of 10 min), the period of oscillations in both experimental groups (Figure 1).

**Figure 2:**
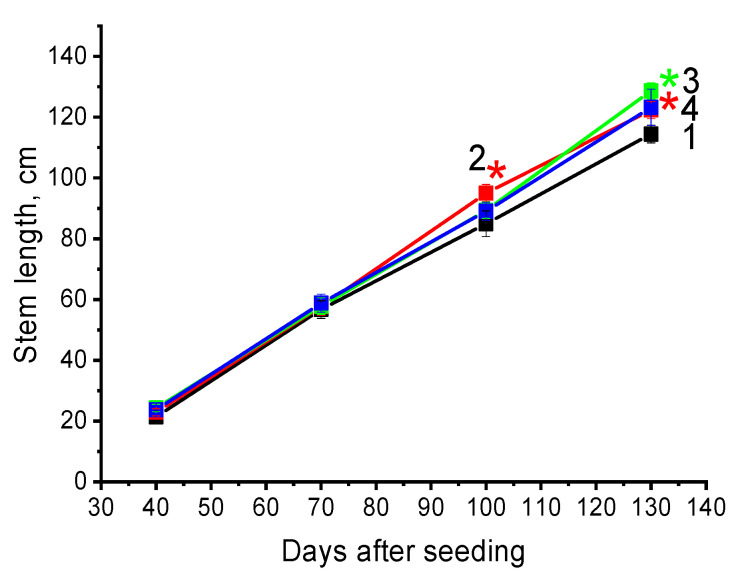
Effect of cotton seeds treatment with pure water (1—black curve) or solution containing 1% PAW (2—red curve), 0.75% PAW (3—green curve) or 0.5% PAW (4—blue curve) for 10 min at room temperature on cotton plant growth in the field. Stars indicate statistically significant difference between the experimental (2, 3 or 4) and control groups (1) of seed (*p* ≤ 0.05). The data are the means of 40 measurements, with the standard deviation of the mean. Experiment were repeated 4 times.

However, under field conditions, pre-soaking of seeds in PALs had a positive effect on cotton stem growth (≈10%) (Table 2, Figure 2). While the stem length of the control plants on the 130th day after germination was 114 cm, then PAW_1%_ plants and PAW_0.75%_ plants were 123 cm and 129 cm, respectively. The increase in stem length of PAW_0.5%_ plants was not statistically significant.

It was shown that under field conditions, approximately 40% of all germinated plants survived to the thinning stage, regardless of the type of PALs used for preliminary seed treatment (Table 3). The yield of cotton plants, in contrast, varied depended on the type of preliminary seed treatment. Control plants produced slightly more than 40 centners of raw cotton per hectare. The yield of PAW_0.5%_ plants was approximately 12% higher in comparison to control plants, while the yield of PAW_0.75%_ plants and PAW_1%_ plants increases by 22–24%.

**Figure 3:**
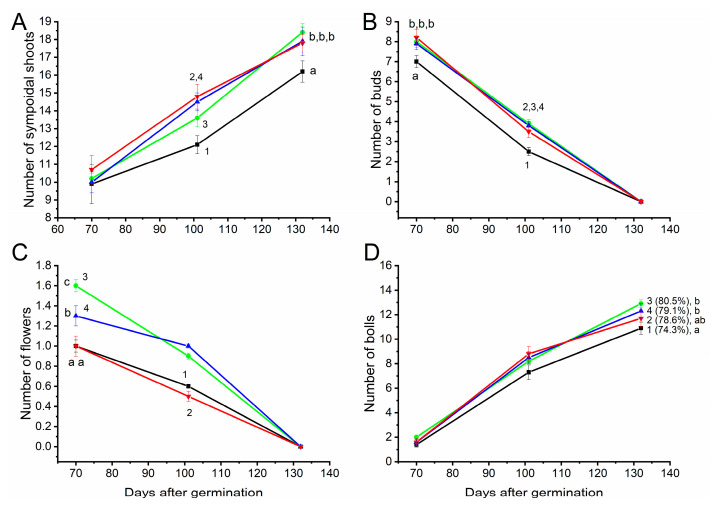
Formation of sympodial branches (**A**), buds (**B**), flowers (**C**), and bolls (**D**) during the growth of cotton plants in the field. Before planting, cotton seeds were incubated in pure water (1—black curve) or solution containing 0.5% PAW (2—red curve), 0.75% PAW (3—green curve), or 1% PAW (4—blue curve) for 10 min at room temperature. The numbers in parentheses in (**D**) indicate the proportion of bolls that opened at the end of the growing season. Letters indicate statistically significant difference between different groups (*p* ≤ 0.05). The data are the means of 40 measurements with the standard deviation of the mean.

It is known that sympodial shoots, buds, flowers and bolls are formed on cotton plants during their growth and development. The analysis showed that plants whose seeds were treated with PAW, on average, formed more sympodial shoots (16 in control plants compared to 18 in experimental plants) (Figure 3A). The number of buds and flowers observed simultaneously in plants was also higher in PAW plants (Figure 3B,C). The number of bolls with seeds formed in PAW_0.75%_ plants was higher in comparison with control plants (Figure 3D), while in PAW_0.5%_ plants and PAW_1%_ plants, statistically significant differences from control plants were not observed. At the same time, the proportion of opened bolls was noticeably higher in the experimental plants (74% in control plants versus ≈80% in experimental plants).

Thus, the pre-treatment of seeds with PAWs has a positive effect on the development of cotton plants, significantly increasing crop yield.

### 2.3. Effect of PAL Added to Nutrition Solution on Cotton Plant Growth and Development

It was shown that when cotton plants were grown under laboratory conditions without PAL in the nutrient solution, the stem length was 21–22 cm on the 32nd day after germination (Table 4). The addition of PAL to the nutrient solution did not affect stem length. However, as shown in Figure 1, a positive effect of PAW was observed when cotton plants (the seeds of which were treated with PAW) were grown in the field. Indeed, the figure shows that the positive effect of PAW is manifested only after several months of cultivation in the field. In addition, a positive effect of PAW was observed when unfavorable conditions (such as water deficiency) were created in the laboratory experiment. While the stem length of control plants decreased to 13.9 cm, PAW_1%_ plants and PAW_0.75%_ plants decreased to 15.3 cm and 15.1 cm, respectively (Table 4).

**Figure 4:**
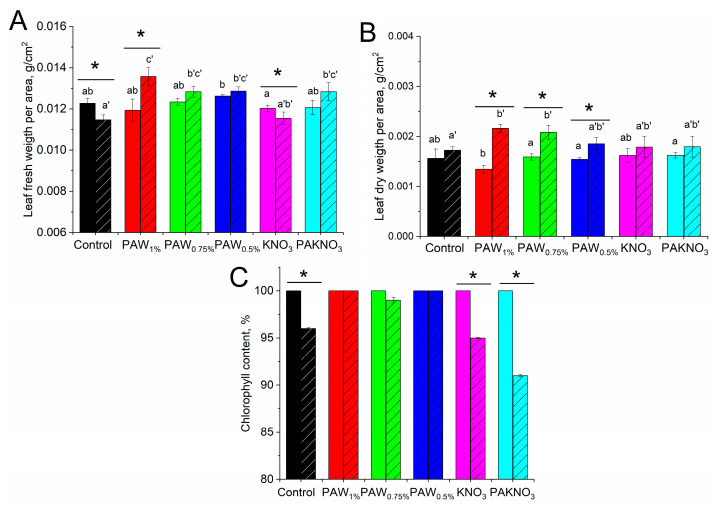
Effect of water deficit on FW/A (**A**), DW/A (**B**) and chlorophyll content (**C**) in cotton leaves of the cotton in the laboratory. The chlorophyll content in cotton leaves not exposed to drought was taken as 100%, which was 9 r. u. (measured by CL-01), which corresponds to 4.05 mg Chl/g FW (for other details, see Section 4). Plants were grown under normal (unshaded columns) and water deficit (shaded columns) conditions and were watered with a nutrient solution without (control plants and KNO_3_ plants) or with a PAL. The designations of plant group below the columns correspond to the designations of the corresponding plant groups in the text. Letters a and b above the columns indicate statistically significant differences between the groups of plants grown under normal conditions (*p* ≤ 0.05). Letters a’, b’ and c’ indicate statistically significant differences between the groups of plants grown under drought conditions (*p* ≤ 0.05). * indicate statistically significant effect of water deficit on plants (*p* ≤ 0.05). The data are the means of 30 measurements with the standard deviation of the mean for FW and DW, and 150 measurements with the standard deviation of the mean for chlorophyll content.

The relative leaf fresh weight (FW/A, calculated as the ratio of the leaf fresh weight to its area) of the control cotton plants decreased slightly (≈7%) under drought conditions likely due to water loss by the leaves (Figure 4A). In the PAL plants, a decrease in FW/A under drought was not observed, and in the case of PAW_1%_ plants, it even slightly increased. Meanwhile, drought did not lead to a change in the relative leaf dry weight (DW/A, calculated as the ratio of the leaf dry weight to its area) in the control plants, as well as in the KNO_3_ plants and PAKNO_3_ plants (Figure 4B). Unexpectedly, the PAW plants responded to water deficit with an increase in DW/A. The increase in DW/A in the leaves of these plants ranged from 20% to 60%, which was in sharp contrast to the response of the control plants. The change in relative leaf weight under water deficit was accompanied by a decrease in chlorophyll content in the control plants. However, in PAW plants, in contrast to PAKNO_3_ plants, such a decrease is not observed (Figure 4C).

When studying the effect of PALs on the root system of cotton plants, it was shown that under both optimal and dry growing conditions, PALs did not affect either the total root length or the general root parameters (Table 4). However, it should be noted that under laboratory conditions, with limited pot size and nutrient solution watering, plants do not need a robust root system, which may explain the lack of statistically significant differences between the plant groups.

### 2.4. Effect of PAL on Photosynthetic Activity of Cotton Plants

**Figure 5:**
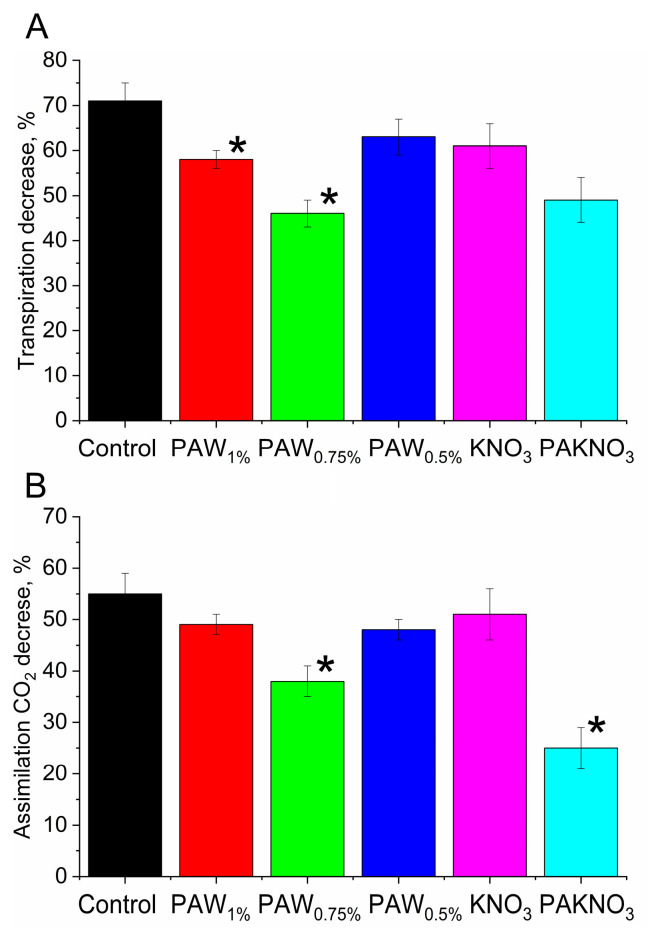
Effect of water deficit on the transpiration intensity (**A**) and the CO_2_ assimilation intensity (**B**) in the leaves of cotton plants grown in laboratory conditions (for more details, see Section 4) watered with a nutrient solution containing no (control plants and KNO_3_ plants) and containing PAL. The designations of the plant groups under the columns correspond to the designations of the corresponding plant groups in the text. The plants were pre-adapted to light for 4 h. The measurements were carried out at 27 °C, 40% humidity and CO_2_ concentration of 400 ppm and acting light λ = 625 nm, 600 µmol photons m^−2^ s^−1^. The rates of CO_2_ assimilation and transpiration for plants of each group grown under normal conditions are taken as 100%. * indicate statistically significant difference between effect of water deficit in PAL plants and the corresponding control plants (*p* ≤ 0.05). The data are the means of eight measurements, with the standard deviation of the mean.

The effect of PALs on the photosynthetic activity of plants grown under laboratory conditions was studied. The intensity of CO_2_ assimilation and the intensity of transpiration in plants did not vary between group of plants and were 8 µmol CO_2_ m^−2^ s^−1^ and 3 mmol H_2_O m^−2^ s^−1^. It was shown that both the intensity of CO_2_ assimilation and the intensity of transpiration decrease under water deficit conditions. The intensity of transpiration decreased by 60–70%, and the intensity of CO_2_ assimilation decreased by 51–55% in control plants and KNO_3_ plants (Figure 5). At the same time, PAL plants under water deficit conditions also reduced the intensity of CO_2_ assimilation and the intensity of transpiration, but these reductions were significantly less than in the control plants. So, the transpiration intensity decreased by ≈50% in PAKNO_3_ plants and by 45–60% in PAW plants. The decrease in the CO_2_ assimilation intensity ranged from ≈25% in PAKNO_3_ plants to ≈40–50% (PAW plants).

**Figure 6:**
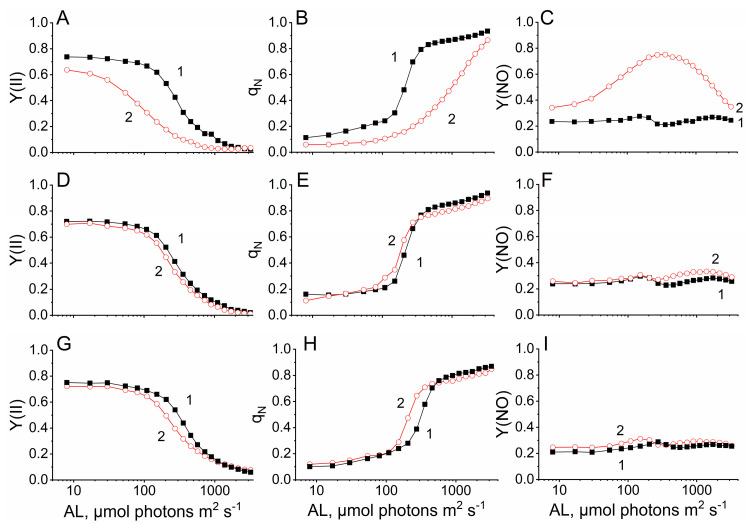
Dependence of the parameters ChlF Y(II) (**A**,**D**,**G**), q_N_ (**B**,**E**,**H**) and Y(NO) (**C**,**F**,**I**) in cotton plants (control plants (**A**,**B**,**C**), PAW_0.75%_ plants (**D**,**E**,**F**) and PAKNO_3_ plants (**G**,**H**,**I**)), grown under normal conditions (curve 1) and under water deficit conditions (curve 2) on the intensity of the acting light during light adaptation of cotton plants grown in laboratory. Measurements were carried out at 27 °C, 40% humidity and CO_2_ concentration of 400 ppm. Plant adaptation to each intensity of acting light (λ = 625 nm) was 10 min. The data are the mean of three measurements with standard deviations which are smaller than the size of the symbols.

Measurement of kinetics of photoinduced changes of the chlorophyll *a* fluorescence yield (ChlF) associated with photoaccumulation of the reduced primary quinone acceptor Q_A_ in photosystem II (F_V_) allowed us to identify some features of the functioning of the photosynthetic apparatus of cotton plants. It was shown that PAL did not affect the maximum quantum yield of chlorophyll *a* fluorescence (F_V_/F_M_) in all plant groups, which was about 0.8. Next, the dependence of ChlF parameters on the intensity of active light was studied in plants adapted to light for 10 min. It was shown that at low light intensity (from 8 µmol photons m^2^ s^−1^ to ≈100 µmol photons m^2^ s^−1^), plants demonstrated a high level of effective quantum yield of photosystem II photochemistry (Y(II)) (Figure 6A). With an increase in light intensity from ≈100 µmol photons m^2^ s^−1^ to ≈700 µmol photons m^2^ s^−1^, a rapid decrease in Y(II) to ≈0.1 was observed, indicating a decrease in the proportion of absorbed light energy directed to the dark stages of photosynthesis. At very high light intensities, a slow decrease in Y(II) (practically to zero) was observed. Watering cotton plants with a solution containing PAL did not change the character of the light curve (Figure 6B,C). However, PAW plants were characterized by a flatter light curve and had higher Y(II) values at high light intensities (Figure 6B). Water deficit changed the nature of the Y(II) light curve. Plants grown under water deficit conditions already had reduced Y(II) values (≈0.65) under weak illumination (Figure 6A). Even a minimal increase in light intensity resulted in a decrease in Y(II), but not as fast as in plants grown under normal conditions, indicating the dominance of another mechanism for reduction of Y(II). Nevertheless, already at 380 µmol photons m^2^ s^−1^, Y(II) reaches minimum values. In the case of PAL plants, water deficit did not affect the Y(II) light curve as strongly. A more rapid decrease in the parameter was observed at light intensities from ≈100 µmol photons m^2^ s^−1^ to ≈700 µmol photons m^2^ s^−1^ in comparison to plants grown under normal conditions (Figure 6D,G). Another way of utilizing the energy of absorbed light is thermal dissipation. Thermal dissipation can occur as a result of both regulatory and unregulated processes. The parameter of non-photochemical fluorescence quenching q_N_ reflects the combined action of two regulatory processes (q_E_—fast quenching regulated by the xanthophyll cycle and triggered by a rising trans thylakoid pH gradient, and q_T_—quenching due to redistribution of excitation energy between PSI and PSII) and photoinhibition. Another parameter Y(NO) reflects the utilization of the energy of absorbed light by unregulated thermal dissipation and fluorescence emission. It was shown that with increasing illumination intensity, a decrease in Y(II) is accompanied by an increase in q_N_ (Figure 6B). As in the case of Y(II), q_N_ developed in three phases. During the first (up to ≈200 µmol photons m^2^ s^−1^) and third (from ≈360 µmol photons m^2^ s^−1^) phases, a gradual increase in the parameter value occurred; in the second phase (from ≈100 µmol photons m^2^ s^−1^ to ≈360 µmol photons m^2^ s^−1^), a sharp increase in the parameter value was observed, probably due to the activation of the xanthophyll cycle.

In control plants grown under water deficit conditions, non-photochemical quenching of chlorophyll *a* fluorescence under weak light was less than in plants grown under normal conditions (Figure 6B). With increasing illumination intensity, q_N_ increased gradually, and the second (fast) phase of parameter growth (characteristic of plants grown under normal conditions) was absent. At the same time, with increasing illumination intensity, non-photochemical quenching increased, probably as a result of photoinactivation of the photosynthetic apparatus. Figure 6C (curve 2) shows that in these plants, the mechanisms of regulated thermal dissipation are replaced by mechanisms of unregulated thermal dissipation, which leads to a drastic increase in Y(NO), which reaches a maximum at ≈360 µmol photons m^2^ s^−1^ and then gradually decreases to the level observed under weak light. Thus, the q_N_ data may indicate that a severe drought stress developed in the control plants. The character of the q_N_ curve of PAW_0.75%_ plants and PAKNO_3_ plants exposed to drought did not change so much. In these plants, the rapid phase of q_N_ development begins (80–100 µmol photons m^2^ s^−1^) and ends (≈280 µmol photons m^2^ s^−1^) somewhat earlier compared to plants not experiencing a water deficit. The Y(NO) parameter in all groups of plants grown under normal conditions was about 0.2, practically not changing with the increase in the illumination intensity. Minor changes in Y(NO) in the light curve (Figure 6C,F,I) can be caused by the change in the efficiency of competing processes reflected in Y(II) and q_N_. Despite the fact that in control plants, water deficit led to a significant increase in Y(NO), in PAW_0.75%_ plants and PAKNO_3_ plants drought stress conditions did not lead to an increase in Y(NO). Note that the nature of the light curves of the parameters shown in Figure 6 in KNO_3_ plants did not differ from those in control plants, and in PAW_0.5%_ plants and PAW_1%_ plants it practically did not differ from those in PAW_0.75%_ plants. Thus, it has been shown that under water deficit conditions, PALs added to the nutrient solution affect the utilization of absorbed light energy by plants. As mentioned above, non-photochemical quenching of chlorophyll *a* fluorescence is a combination of at least three processes: q_E_, high-energy state fluorescence quenching; q_T_, the so-called “state transition”; and q_I_, fluorescence quenching induced by photoinactivation of photosynthetic apparatus. It has been shown that in control plants grown under normal conditions at high illumination intensity (600 µmol photons m^2^ s^−1^), the dominant component of non-photochemical quenching of ChlF is q_E_ (0.68), and the q_I_ value is approximately two times lower (0.35) (Table 5). Note that q_T_ was negligibly small in all plants, probably due to the fact that the measurements were carried out at high illumination intensity.

The contribution of q_E_, q_I_ and q_T_ to the total q_N_ in plants watered with a nutrient solution containing PAL did not differ from the contribution of q_E_, q_I_ and q_T_ measured for the control plants. In the control plants, as well as in the KNO_3_ plants grown under water deficit, a decrease in the q_N_ parameter was observed. The decrease in the q_N_ parameter was due to a significant (≈85%) decrease in q_E_ that may indicate a high level of stress. At the same time, q_I_ practically did not increase. In PAW plants and PAKNO_3_ plants, water deficit practically did not affect the total level of non-photochemical quenching of ChlF that may indicate an increase in drought tolerance of plants watered with PAL. However, in some groups of plants, the q_N_ structure changed. In PAKNO_3_ plants, water deficit caused a 15% decrease in q_E_, in contrast to the other groups of PAL-treated plants. PAW_0.75%_ plants significantly decreased q_I_, which indicates a slowdown in the processes of photoinactivation of the photosynthetic apparatus of plants in this plant group. Thus, the treatment of plants with PAL leads to a decrease in the negative impact of water deficiency, which manifests itself in the disruption of the partitioning of absorbed light energy and photoinactivation of the photosynthetic apparatus of the studied plants.

## 3. Discussion

### 3.1. Parameters of Plasma-Activated Water

It is known that during plasma treatment of liquids, a whole cascade of oxidation-reduction reactions occurs, leading to the formation of various active forms of nitrogen, active forms of oxygen, etc. However, most of these species are short-lived, which makes it impossible for them to influence biological objects as part of PAL (except for direct treatment of biological objects, for example, with cold plasma) [25]. Therefore, researchers mainly take into account long-lived forms that can accumulate during the preparation of PAL and persist for many hours and even weeks. As shown earlier, PAW accumulates long-lived biologically active compounds such as hydrogen peroxide, NO_2_^−^, NO_3_^−^, peroxynitrite, etc. during the preparation process [10,24,26].

As shown in Table S1, the two kinds of PALs obtained in our study differed in their physicochemical properties. The main difference was observed in the pH value and the concentration of long-lived biologically active species of nitrogen and oxygen. PAW had a pH of 5.6 while the pH of PAKNO_3_ reached 10.8. However, the initial pH of PAL could not exert a significant effect on cotton seeds and plants because the dilution of PAL in the obtained solutions adjusted the pH to that of deionized water (pH 6.7–6.8). The concentration of hydrogen peroxide in PAW was significantly higher than that in PAKNO_3_ (150 μM and 1.1 μM, respectively), while the content of NO_2_^−^ and NO_3_^−^ was higher in PAKNO_3_ (300 μM and 1500 μM in total, respectively). So, the PALs received in the present work have similar characteristics with those received earlier [27,28]. It should be noted that the measurements of the PAL properties were performed in the same time interval after preparation, in which it was diluted for further treatment of seeds or plants. It was shown that the concentration of biologically active substances did not undergo statistically significant changes in the time period required for the treatment of seeds and plants (from 30 to 60 min after PAL preparation). The half-life of hydrogen peroxide in PAW was more than 7 days, while in PAKNO_3_ H_2_O_2_ disappears within 24 h after preparation. In earlier studies, it was shown that, depending on the plasma source, the type of activated solution, the working atmosphere, etc., the half-life of hydrogen peroxide in PAL can range from 8 h to 20 [10,29]. At the same time, the addition of PAL to biological objects, such as plant seeds, leads to the rapid disappearance of hydrogen peroxide [30]. Conversely, low concentrations, the absence of transition metal ions and a substrate for interaction prolong the existence of peroxide in PAL. In our case, the already not very high concentrations of hydrogen peroxide and RNS were reduced by two orders of magnitude, which only increased the stability of these compounds in PAL. Many studies have shown that the RNS content also remains virtually unchanged over a long period of time during PAL storage. However, the presence of biological objects, as in the case of hydrogen peroxide, leads to rapid consumption of both NO_2_^−^ and NO_3_^−^ [30]. In many cases, the relatively slow aging of PAL allows for the treatment of biological objects long after PAL preparation [30].

### 3.2. Effect of PAL on Seed Germination and Plant Growth Under “Ideal” Conditions

An analysis of the literature data shows that many researchers have not observed PAL-induced stimulation of plant growth and seed germination. This applies to those cases (and the present study is no exception) when the researchers either used very high-quality seeds or grew plants under “ideal” conditions. The authors’ work showed that PAL did not increase the germination of *Lactuca Sativa* L. seeds, which have a germination rate of about 95% [31]. PAL treatment of corn seeds did not lead to a significant difference in germination compared to control seeds [32]. The authors suggested that the lack of effect was due to the high percentage of seed germination even without treatment with PAL. In the work [30], the authors used high-quality wheat seeds (95–96% natural germination) and found that PAL created from deionized water did not affect germination. However, the germination time of PAL-treated seeds was slightly reduced compared to the control. Our experiments show that the application of PAL under “ideal” conditions, which certainly include laboratory conditions, does not increase the productivity of cotton plants. These data may evidence that PAL added in the indicated concentrations does not have a negative effect on either plants or seeds. As in the case of high-quality seeds, plants growing under optimal conditions did not respond to PAL treatment, since the plants had no limitations in mineral nutrition and amount of light, and were not exposed to stress factors such as temperature, drought, salinity, etc. At the same time, the effect of PAL is usually associated with the action of its biologically active species on both the plant itself and the surrounding microflora, as well as the presence in the activated liquids of a nitrogen source that can be absorbed by plants and included in metabolism. Previously, Kučerová and co-authors showed that the effect of PAL on lettuce plant growth under laboratory conditions was very limited compared to the effect of a chemically equivalent solution of H_2_O_2_ + NO_3_^−^: no effect of PAL on the total dry weight of above-ground parts and roots was detected [14]. Another study showed that the effect of PAL on plant sprout development is not always positive [33], an excess of long-lived products in plasma-treated liquid can negatively affect the development of mung bean sprouts.

### 3.3. Effect of PAL on Seed Emergence and Plant Growth Under Stress Conditions

Despite the lack of a stimulating effect of PAL on plant growth in the laboratory, a significant positive effect was found in the field. Obviously, conditions in the laboratory and in the field are very different. The positive effect of PAW in the field may be attributed, on the one hand, to the extended duration of the experiment (compared to laboratory conditions) and, on the other hand, to the impact of unfavorable environmental factors in open-ground conditions, that humans cannot influence. It is not surprising that seed priming with PAL had such a beneficial effect on field germination and further plant development. It is known that a number of factors affect seeds during PAL treatment. Among them are such effects as sterilization, modification of the seed surface, activation of signaling pathways by active forms of nitrogen and oxygen, etc. It was previously shown that soaking seeds in PAL generated in a plasma reactor based on a single-electrode atmospheric-pressure plasma jet increased the yield of *Lactuca sativa* L. both in the field and in the greenhouse [34]. However, a positive effect of PAL, which we found in the field, was repeated in the laboratory when plants were grown under water deficit. It is known that significant losses in both biomass and quality of agricultural products occur under of a constantly changing environment conditions, as well as under the influence of biotic factors. In addition to improving plant growth, PAL activated the germination of pre-aged seeds by heat treatment. Under these conditions, the signaling pathways responsible for seed dormancy may be damaged in seeds. Many authors note that PAL is able to increase the germination of seeds of poor quality. It was previously shown that seed treatment with cold plasma improved the germination of soybean seeds under conditions of water deficit and affect the antioxidative system of barley (*Hordeum vulgare*) on a long-term scale [35,36]. In addition, the positive effect of plasma treatment of seeds was shown under normal conditions [37,38], while the opposite effect was also found [39]. The authors suggest that the main activating agents under such treatment are reactive oxygen and nitrogen species (RONS), since it was previously noted that RONS can be involved in the germination process. In this case, the possibility of participation of both endogenous (for example, produced by mitochondria) and exogenous (from PAL) ROS is especially emphasized [40]. Activation of seed germination by PAL containing hydrogen peroxide was found in *Paulownia tomentosa*. In this case, the activation mechanism is assumed to be similar to that found in seeds treated with a hydrogen peroxide solution [41]. Similar mechanisms of seed germination activation are assumed for RNS formed in PAL [42]. In addition, our experiments showed that seed germination is improved by adding both non-activated and activated KNO_3_. It is known that NO_3_, including KNO_3_, stimulate the germination of plant seeds [43]. A similar effect can be exerted by NO_3_ formed in PAW. In addition to all of the above, PAL can increase the percentage of germination due to the sterilizing effect [10,44].

In our work, it is shown that PALs have a positive effect not only on seed germination, but also on subsequent plant development, and also as a component of the nutrient solution. The positive effect of soybean seed treatment with cold plasma on plant development was discovered earlier [36]. In turn, PAL treatment of rye and radish seeds led to an increase in root length and plant height [45]. Moreover, the positive effect of seed treatment with PAL on plant growth was also shown using sorghum, strawberry and spruce [46]. It is assumed that such an indirect effect, when the seed is treated, but the growth of the whole plant is stimulated, may be due to ROS-dependent production of hormones by the seed and activation of the intracellular signaling system [15,45]. One of the main reasons of the positive effect of PAL, as a component of the nutrient solution, is the ability of plants to absorb nitrogen species contained in activated liquids through the roots and include them in metabolic pathways [47,48,49]. However, in our experiments, the plants were grown with sufficient nitrogen nutrition, and PALs themselves, diluted 100–200 times, did not increase the nitrogen content in the nutrient solution. In addition to RNS, the presence of hydrogen peroxide is the cause of the activating effect of PAL added to the nutrient solution [49,50]. As shown in the present work, PAW, accumulating up to 150 µM H_2_O_2_, had a more pronounced positive effect on cotton plants (only under drought conditions) in comparison to PAKNO_3_, which contains two orders of magnitude less hydrogen peroxide. The data presented in Figure 4 may indicate that the loss of water by the leaves in the control plant groups and PAKNO_3_ plants is accompanied by a nearly proportional inhibition of photosynthetic capacity. A watering of the plants with PAW somehow helped prevent the loss of photosynthetic capacity in the leaves of the plants. It is consistent with a decrease in drought-induced inhibition of CO_2_ assimilation intensity in PAW-treated plants. Nevertheless, a positive effect of PAKNO_3_ on the intensity of CO_2_ assimilation under drought stress also appeared.

Chlorophyll *a* fluorescence data indicate that PALs, in general, did not change the efficiency of the photochemistry in the plants that growing under normal conditions. However, under drought conditions, PALs reduced the negative effect of water deficit on redistribution of absorbed light energy (Figure 6). The data indicate that control plants develop severe stress, while PAL-treated plants develop moderate stress. It is known that during drought, plants become more sensitive to the inhibitory effect of light; therefore, mechanisms are launched in plants that enhance the dissipation of excitation energy into heat, which ultimately leads to an increase in q_N_ [51]. And only severe drought stress leads to the loss of the ability to regulate the dissipation of excitation energy [51]. Indeed, the data shown in Table 2 indicate a reduction in drought-mediated photoinhibition of the photosynthetic apparatus in PAW plants.

It was previously found that during plasma treatment, platinum nanoparticles accumulate in the PAKNO_3_ solution, which, according to the authors, can have a positive effect on plant growth. Another study showed that the presence of up to 10 mg/L of platinum nanoparticles (5 nm) in the nutrient solution increased the length and weight of roots, as well as the flavonoid content in the leaves of hydroponically grown wheat [52]. Thus, the differences in the effect of the two types of PAL, obtained by different methods, on plants may be due to the different contents of long-lived reactive oxygen species.

## 4. Materials and Methods

### 4.1. Production and Characterization of PAL

**Figure 7:**
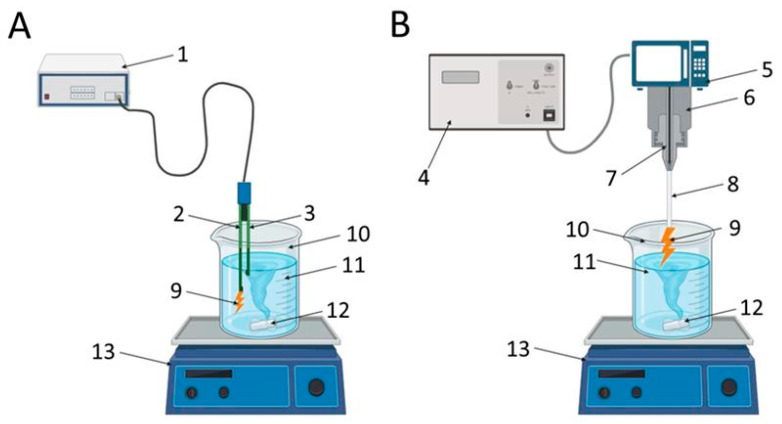
Block diagrams of installations for obtaining plasma-activated solutions. Activation was carried out using plasma generated in an electrochemical cell (**A**), generated by a microwave plasmatron (**B**). 1—generator, 2—cathode, 3—anode, 4—power source, 5—magnetron, 6—external electrode, 7—internal electrode, 8—quartz capillary, 9—plasma discharge, 10—glass beaker, 11—activated liquid (deionized water in (**A**) or electrolyte solution in (**B**)), 12—magnetic mixer, 13—magnetic stirrer.

Plasma-activated solutions were produced using two approaches: by microwave treatment using a magnetron in an ambient air atmosphere [23] and by using a glow discharge in an electrochemical cell without a diaphragm [53]. In the first case, a pulse duration of 105 ms with a frequency of 50 Hz and a power of 2 kW was used in the magnetron. The average microwave power was 10.5 W, and the specific energy input per 1 cm^3^ of water was 189 J/cm^3^. In the second case, in the electrochemical cell, a voltage of 200–300 V was applied to the active platinum electrode from a generator operating at a frequency of 440 kHz. As a result of the contact between the electrode and the solution, plasma formation occurred. In both cases, the solutions were treated for an hour with constant stirring (Figure 7). Plants and seeds were treated within 30–60 min after PAL preparation, since delivering of PAL to the place where the plants were grown and to prepare solutions for watering/treating took about 30 min. At the same time, treatment of all plants/seeds took no more than 30 min. Preliminary experiments showed that the physicochemical parameters of the surfactant remained virtually unchanged during this time.

The concentrations of H_2_O_2_ and NO_2_^−^ ions were determined spectrophotometrically using HACH LANGE DR-5000 spectrophotometer (HACH LANGE GmbH, Düsseldorf, Germany) [54,55]. NO_3_^−^ ions were detected using LAQUAtwin NO3-11 (HORIBA Advanced Techno, Kyoto, Japan). The conductivity, pH, and redox potential of the liquids were determined using a SevenExcellence multichannel meter (Mettler Toledo, Greifensee, Switzerland).

### 4.2. Seed Germination and Field Emergence

To determine the germination rate of *Gossypium hirsutum* L. (C-5707 variety) seeds, lint seeds were used. A total of 1250 standardized seeds were soaked (for 10 min with constant stirring) in deionized water (control seeds), in a 0.015% KNO_3_ solution (KNO_3_ seeds), in a 0.5% aqueous solution of PAW (PAW_0.5%_ seeds), in a 0.75% aqueous solution of PAW (PAW_0.75%_ seeds), in a 1% aqueous solution of PAW (PAW_1%_ seeds), or in an activated 1.5% KNO_3_ solution diluted 100 times (1.48 mol/L) (PAKNO_3_ seeds). From each group, 250 seeds (previously divided into five independent groups) were left for germination in Petri dishes. The other 1000 seeds (divided into four independent groups of 250 seeds each) were sown in open soil (at the Research Institute of Cotton Breeding, Seed Production, and Agricultural Technology of the Republic of Uzbekistan). It should be noted that field germination was studied only for the control seeds and PAW seeds. The seeding rate of lint seeds in the field was 100,000 seeds (125 kg) per one hectare. Field emergence was recorded on the seventh day.

The seed senescence process imitation was carried out as follows. The seeds were placed in a thermostat at 45 °C and 99% air humidity for 75 h. Then, the seeds were kept for five days at room temperature and 40% air humidity in the dark. After that, the seeds were treated with experimental and control solutions and tested for germination as described above.

### 4.3. Plant Grow Conditions

The plants were grown under laboratory conditions on the special shelves with a 16-h photoperiod and a day/night temperature of 27 °C/17 °C. The intensity of photosynthetically active radiation (λ = 400–700 nm) was 600 μmol photons m^2^ s^−1^. The PG200N spectrometer (UPRtek, Zhunan, Miaoli, Taiwan) was used to measure the light flux density. A soil mix (Miracle garden, Moscow, Russia) combined with vermiculite in a 3:1 ratio was used as a substrate. Watering was carried out every 5 days throughout the experiment, alternating between a nutrient solution and deionized water. The nutrient solution contained 0.05 g/L NH_4_NO_3_; 0.17 g/L KNO_3_; 1.06 g/L Ca(NO_3_)_2_ 4H_2_O; 0.38 g/L K_2_SO_4_; 0.135 g/L KH_2_PO_4_; 0.49 g/L MgSO_4_ 7H_2_O. Water deficit was created by discontinuing watering with deionized water, while maintaining watering with the nutrient solution every 10 days. Analyses and measurements of water-deficient plants were conducted on the ninth day after the previous watering, when the maximum water stress in plants was reached.

The effect of soaking seeds in PAL on plant growth and development was conducted as follows. Soaking of at least 150 seeds in each group was carried out as described in Section 4.2. After soaking, the seeds were placed in 100 mL pots (one seed per pot).

In the study of the effect of PAL as a component of the nutrient solution on plant growth and development, at least 150 seeds (divided into three independent groups of 50 seeds each) were planted in the soil mixture in a 100 mL pot without soaking. To avoid exceeding the concentration of KNO_3_ in the nutrient solution when watering KNO_3_ plants and PAKNO_3_ plants, the KNO_3_ in the nutrient solution was replaced with the corresponding amount of activated and non-activated KNO_3_ solution. It should be noted that the control plants and KNO_3_ plants are the same in this experiment, as their nutrient solutions have identical compositions during watering. However, since different stock solutions were used to prepare the nutrient solutions for these groups, and to adhere to all experimental procedures, these plants were assigned into separate groups.

For the open-field experiment, plants obtained from the field experiment described in Section 4.2 were used and grown under natural conditions. After the formation of two true leaves, thinning was carried out, and before this, the number of surviving plants was manually counted. Throughout the experiment, the number of buds, sympodial shoots, open and unopened cotton bolls was manually recorded. After 130 days of growth, the cotton plant yield was determined.

Field experiments were carried out on the experimental plot of the central experimental farm of the Research Institute of Selection, Seed Growing and Agrotechnology of Cotton Growing. The institute is located at the coordinates 41° north latitude and 69° east longitude, in the village of Salar, Tashkent region. The altitude above sea level is 584 m.

The soil is irrigated gray soil. Groundwater lies at a depth of 7–8 m. The climatic conditions of the region are sharply continental, with a high amplitude of daily temperature fluctuations. The average daily air temperature was +25–26 °C. The average annual precipitation was 360 mm.

### 4.4. Determination of Morphological and Physiological Indicators of Cotton Plants

To determine the ratio of fresh and dry leaf weight to leaf area, leaf discs (diameter = 1 cm) were used, which were weighed before and after drying (for fresh and dry weight, respectively) using an OHAUS MB-27 moisture analyzer (OHAUS CIS, Moscow, Russia).

The root length was determined according to the following protocol. First, the roots with soil were soaked in water at 25 °C for 10 min. Then, the soil was washed off using tweezers and a brush [56]. The cleaned roots were separated into segments (primary and adventitious) to reduce overlap during scanning [57]. The root segments were placed in a transparent tray with room temperature water. Scanning was performed using a stationary HP Scanjet 4850 scanner (HP, Palo Alto, CA, USA) with a resolution of 1200 dpi [56]. The resulting images were analyzed using the RhizoVision Explorer v. 2.0.3 software [58]. The length of the primary root was determined using the image analysis software ”Digimizer v. 6.4.0” (MedCalc Software Ltd, Ostend, Belgium) [59].

Chlorophyll content was monitored using a chlorophyll meter CL-01 (Hansatech Instruments Ltd., Norfolk, UK). To convert the obtained values into generally accepted units of measurement (mg chl g^−1^ of fresh weight), we investigated the dependence of the values obtained using CL-01 on the actual chlorophyll content in the leaves of plants grown under the same conditions as experimental plants. The actual chlorophyll content was measured in fresh leaf samples (0.6 g). Leaf samples were homogenized in ethanol (95% *v*/*v*), left in the dark for 10 min, then filtered and centrifuged for 5 min at 15,000 rpm. The chlorophyll concentration was calculated from the absorbance of the extract at 664 nm and 648 nm using the formula:

*C*_*a*+*b*_ = 5.24 ∗ *A*_(664)_ + 22.24 ∗ *A*_(648)_ (1)

where *A*_(664)_ is the absorption at λ = 664 nm, *A*_(648)_ is the absorption at λ = 648 nm. Based on the obtained data, a calibration curve was built, and a linear dependence was fitted for the calculation of the chlorophyll content into generally accepted units “mg chl g^−1^ of fresh weight” y = 0.15x + 2.72. A calibrated curve was built based on 50 measurements.

### 4.5. Effect of PAL on Photosynthetic Activity of Cotton Plants

The measurements of assimilation of CO_2_ and the transpiration intensity were carried out using the GFS-3000 (Waltz, Eichenring, Effeltrich, Germany) and GFS-win software (v. 3.79) according to Equations (2) and (3) [60]. Plants were pre-adapted to light for 4 h to achieve the steady-state values of gas exchange parameters, and then to conditions in a measuring chamber for 10 min (25 °C, 65% humidity in a laminar CO_2_ flow 400 with concentration 400 ppm, λ = 625 nm, 600 µmol photons s^−1^ m^−2^).

(2) $$E=\frac{\mathrm{Ue}*\left(\mathrm{Wo}-\mathrm{We}\right)}{\mathrm{LA}*\left(1-\mathrm{Wo}\right)},$$

(3) $$A=\frac{\mathrm{Ue}*\left(\mathrm{Ce}-\mathrm{Co}\right)}{\mathrm{LA}}-E*\mathrm{Co},$$

where E—transpiration rate, A—assimilation rate, Ue—molar flow rate at the inlet of the chamber, Wo—H_2_O mole fraction at the outlet of the chamber, We—H_2_O mole fraction at the inlet of the chamber, LA—leaf area (2 cm^2^), Co—CO_2_ mole fraction at the outlet of the chamber, Ce—CO_2_ mole fraction at the inlet of the chamber.

To measure the induction curve of ChlF in the leaves of cotton plants a Multi-Color-PAM fluorometer (Walz, Eichenring, Effeltrich, Germany) was used. Measurements were conducted in a measurement chamber where conditions similar to those in the growth chamber during the light period were maintained (27 °C, 65% humidity in a laminar CO_2_ flow with a concentration of 400 ppm, and actinic light (AL) intensity = 600 µmol photons m^−2^ s^−1^). Before measurements, the plants were adapted to darkness for 1 h for complete relaxation of the all light-induced processes. The plants were adapted to AL for 10 min. The duration of the saturating pulse was 300 ms (λ = 625 nm, 12,000 µmol photons s^−1^ m^−2^). ChlF parameters were calculated using PamWin-3 software according to Equations (4)–(7):

(4) $$\frac{\mathrm{Fv}}{F_{M}}=\frac{F_{M}-\mathrm{Fo}}{F_{M}},$$

(5) $$Y\left(\mathrm{II}\right)=\frac{{F_{M}}^{\prime }-F}{{F_{M}}^{\prime }},$$

(6) $$\mathrm{qN}=\frac{F_{M}-{F_{M}}^{\prime }}{F_{M}-{\mathrm{Fo}}^{\prime }},$$

(7) $$Y\left(\mathrm{NO}\right)=\frac{1}{\mathrm{qN}+1+\frac{{F_{M}}^{\prime }-F}{{F_{M}}^{\prime }-{\mathrm{Fo}}^{\prime }}*\frac{{\mathrm{Fo}}^{\prime }}{F}*\frac{F_{M}}{\mathrm{Fo}-1}},$$

where Fo—the intensity of ChlF caused by measured light, F_M_—the maximal level of ChlF caused by actinic light, F_V_—the photoinduced change in the yield of ChlF, Y(II)—the effective quantum yield of PSII photochemistry, F_M_′—the light-induced maximal level of ChlF in light-adapted leaves, F—the intensity of ChlF measured immediately before a saturated pulse of light, Fo’—minimal ChlF yield of illuminated sample, qN—the coefficient of nonphotochemical quenching, Y(NO)—the quantum yield of nonregulated energy dissipation in PS II.

### 4.6. Statistical Analysis

Statistically significant differences between plant groups were revealed using one-way analysis of variance (ANOVA) with the OriginPro 2018 v. 9.5.1.195 software, followed by post hoc comparison using Tukey’s test and Student’s t-test for independent means. The normality (Shapiro–Wilk test) and homoscedasticity (Goldfeld–Quandt test) requirements were checked. The difference was considered statistically significant if *p* ≤ 0.05. Both the field and laboratory experiments were repeated at least 3 times.

## 5. Conclusions

Thus, we obtained two types of plasma-activated solutions with different compositions of long-lived biologically active elements. Plasma-activated 1.5% KNO_3_ solution contained a large amount of active forms of nitrogen, while plasma-activated water contained a large amount of hydrogen peroxide. We assume that this is the reason for the different effects of the solutions on cotton plants. The first solution had a positive effect on the germination of heat-treated seeds. The second solution not only improved seed germination in field conditions, but also improved the growth of cotton plants.

According to the server https://pubmed.ncbi.nlm.nih.gov/ (accessed on 20 September 2024), five to eight hundred studies are published annually on the production and application of plasma-activated liquids. A significant proportion of these studies describe the effect of PAL on plants. Obviously that PAL can be an effective remedy in agriculture due to its positive effect on both seed material and plants in general, as well as the possibility of application of it as a sterilizing agent in the post-harvest period. The undoubted advantages of PAL include its relative cheapness, ease of application, and almost absolute environmental safety.

## Figures and Tables

**Table 1:** Effect of PAL on the germination of cotton seeds in the laboratory and field emergence.

Experimental Groups	Laboratory Experiment	Field Experiment	Laboratory Experiment Using Artificially-Aged Seeds
Control seeds	100	78.2 ^a^ ± 2.0	83.3 ^a′^ ± 2.1
PAW_0.5%_ seeds	100	83.9 ^b^ ± 2.1	86.7 ^b′^ ± 1.9
PAW_0.75%_ seeds	100	85.7 ^b^ ± 2.1	87.3 ^b′^ ± 2.3
PAW_1%_ seeds	100	79.5 ^a^ ± 2.0	80.0 ^a′^ ± 2.9
KNO_3_ seeds	100	–	90.9 ^b′^ ± 2.0
PAKNO_3_ seeds	100	–	88.4 ^b′^ ± 1.8

Letters indicate statistically significant difference between different seed groups (*p* ≤ 0.05). The data are the means of three replications for laboratory experiment and four replications for field experiment with the standard deviation of the mean.

**Table 2:** Effect of PAL treatment of cotton seeds on cotton plant growth on 24th day and 130 day (for laboratory and field experiment, respectively) after seeding.

Experimental Groups		Laboratory Experiment			Field Experiment
		Stem Length, cm	Total Root Length, cm	General Root Length, cm	Stem Length, cm
Without seed soaking		21.3 *^a^* ± 0.9	563 *^a′^* ± 137	21.8 *^a″^* ± 1.5	–
After seed soaking	Control plants	22.1 *^a^* ± 1.1	588 *^a′^* ± 36	21.8 *^a″^* ± 3.2	114 *^a‴^* ± 5.9
	PAW_0.5%_ plants	21.4 *^a^* ± 0.8	573 *^a′^* ± 51	23.2 *^a″^* ± 2.9	122 *^a‴, b‴^* ± 6.1
	PAW_0.75%_ plants	22.0 *^a^* ± 0.9	564 *^a′^* ± 59	24.6 *^a″^* ± 2.4	129 *^b‴^* ± 6.4
	PAW_1%_ plants	20.9 *^a^* ± 0.5	520 *^a′^* ± 75	24.7 *^a″^* ± 1.9	123 *^b‴^* ± 6.2
	KNO_3_ plants	21.3 *^a^* ± 1.0	490 *^a′^* ± 92	22.3 *^a″^* ± 2.8	–
	PAKNO_3_ plants	22.6 *^a^* ± 1.1	527 *^a′^* ± 71	23.3 *^a″^* ± 2.7	–

Letters indicate statistically significant difference between different plant groups (*p* ≤ 0.05). The data of laboratory experiment are the means of at least 9 or 30 measurements with the standard deviation of the mean for root length and stem length, respectively. The data of field experiment are the means of at least 40 measurements with the standard deviation.

**Table 3:** Effect of PALs on plant survival and yield of raw cotton in the field.

Experimental Groups	Number of Surviving Plants, Thousands Per One ha	Number of Plants After Thinning, Thousands Per One ha	Raw Cotton Yield, Centners Per	
			One ha	Per Thousand Plants
Control plants	326 *^a^* ± 16	65.2 *^a^* ± 2.8	40.8 *^a^* ± 2.0	0.626 *^a^* ± 0.031
PAW_0.5%_ plants	331 *^a^* ± 12	66.3 *^a^* ± 2.7	45.5 *^b^* ± 2.1	0.686 *^b^* ± 0.032
PAW_0.75%_ plants	357 *^b^* ± 17	71.4 *^b^* ± 3.1	50.6 *^c^* ± 1.5	0.709 *^c^* ± 0.021
PAW_1%_ plants	350 *^ab^* ± 15	69.9 *^ab^* ± 3.1	49.9 *^c^* ± 2.2	0.714 *^c^* ± 0.031

The data are the means of four replications with the standard deviation of the mean. Letters indicate statistically significant difference between different plant groups (*p* ≤ 0.05).

**Table 4:** Effect of PAL added in nutrition solution on cotton plant growth in the laboratory.

Experimental Groups		Stem Length, cm	Total Root Length, cm	General Root Length, cm
Control plants	N	21.3 *^a^* ± 0.9	563 *^a′^* ± 137	21.8 *^a″^* ± 1.5
	D	13.9 *^b^* ± 0.6	570 *^a′^* ± 26	23.3 *^a″^* ± 0.7
PAW_0.5%_ plants	N	21.2 *^a^* ± 0.4	494 *^a′^* ± 194	25.2 *^a″^* ± 4.7
	D	14.0 *^b^* ± 0.6	541 *^a′^* ± 48	19.6 *^a″^* ± 2.8
PAW_0.75%_ plants	N	20.8 *^a^* ± 0.6	516 *^a′^* ± 17	18.8 *^a″^* ± 3.7
	D	15.1 *^c^* ± 0.3	620 *^a′^* ± 81	23.1 *^a″^* ± 1.4
PAW_1%_ plants	N	21.7 *^a^* ± 0.6	574 *^a′^* ± 135	19.4 *^a″^* ± 2.3
	D	15.3 *^c^* ± 0.6	541 *^a′^* ± 160	25.1 *^a″^* ± 1.6
KNO_3_ plants	N	22.1 *^a^* ± 2.7	481 *^a′^* ± 42	25.8 *^a″^* ± 2.0
	D	13.7 *^b^* ± 1.2	573 *^a′^* ± 67	22.9 *^a″^* ± 3.4
PAKNO_3_ plants	N	19.6 *^a^* ± 3.4	528 *^a′^* ± 137	28.6 *^a″^* ± 1.2
	D	13.7 *^b^* ± 1.3	667 *^a″^* ± 40	25.4 *^a″^* ± 2.2

Letters indicate statistically significant difference between different seed groups (*p* ≤ 0.05). N and D indicate normal and drought growing conditions. The data are the means of at least 9 or 30 measurements with the standard deviation of the mean for root length and stem length, respectively.

**Table 5:** Effect of PAL added to nutrition solution on light-induced non-photochemical quenching of ChlF in cotton plants grown in the laboratory.

Experimental Groups		q_N_	q_E_	q_I_	q_T_
Control plants	N	0.797 *^a,b^* ± 0.023	0.675 *^a′b′^* ± 0.031	0.350 *^a″^^b^^″^* ± 0.032	0.038 ± 0.027
	D	0.470 *^c^* ± 0.016	0. 108 *^c′^* ± 0.020	0.376 *^a^^″^* ± 0.031	0.052 ± 0.032
PAW_0.5%_ plants	N	0.789 *^a,b^* ± 0.007	0.679 *^a′b′^* ± 0.031	0.302 *^b^^″^* ± 0.002	0.057 ± 0.055
	D	0.773 *^a^* ± 0.022	0.654 *^a′b′^* ± 0.046	0.335 *^a″b″^* ± 0.025	0.014 ± 0.008
PAW_0.75%_ plants	N	0.815 *^b^* ± 0.015	0.699 *^a′b′^* ± 0.023	0.374 *^a^^″^* ± 0.023	0.039 ± 0.021
	D	0.792 *^a,b^* ± 0.018	0.677 *^a′b′^* ± 0.030	0.328 *^b″^* ± 0.015	0.040 ± 0.019
PAW_1%_ plants	N	0.803 *^a,b^* ± 0.116	0.678 *^a′b′^* ± 0.170	0.322 *^a″^^b^^″^* ± 0.084	0.098 ± 0.035
	D	0.826 *^b^* ± 0.027	0.722 *^a′^* ± 0.040	0.331 *^b″^* ± 0.002	0.063 ± 0.019
KNO_3_ plants	N	0.800 *^a,b^* ± 0.022	0.657 *^a′b′^* ± 0.058	0.364 *^a″^^b^^″^* ± 0.036	0.080 ± 0.020
	D	0.489 *^c^* ± 0.035	0.105 *^c′^* ± 0.029	0.387 *^a^^″b^^″^* ± 0.056	0.077 ± 0.034
PAKNO_3_ plants	N	0.816 *^b^* ± 0.006	0.714 *^a′^* ± 0.019	0.324 *^b^^″^* ± 0.024	0.046 ± 0.020
	D	0.784 *^a,b^* ± 0.046	0.609 *^b′^* ± 0.070	0.347 *^a″b″^* ± 0.030	0.035 ± 0.039

Letters indicate statistically significant difference between different seed groups (*p* ≤ 0.05). N and D indicate normal and drought growing conditions. The data are the means of three measurements with the standard deviation of the mean.

## Data Availability

The data presented in this study are available on request from the corresponding author.

## Supplementary Materials

The following supporting information can be downloaded at: https://www.mdpi.com/article/10.3390/plants14030304/s1, Figure S1: Representative kinetics of photoinduced changes of chlorophyll fluorescence yield (F_v_) related to photoreduction of the primary electron acceptor Q_A_. F_0_—the level of fluorescence induced by the measuring light; F_m_—the level of fluorescence induced by a single saturating pulse in dark-adapted (60 min) samples, ↑—a single 200 ms saturating flash (λ = 625 nm, 12,000 μmol photon s^−1^ m^−2^), then shut down actinic light (AL), then inclusion far red light (FRL), then shut down (FRL) and inclusion AL. Plant adaptation to each intensity of AL (λ = 625 nm) was 10 minutes. Measurements were carried out at 27 °C, 40% humidity and CO_2_ concentration of 400 ppm and repeated 3 times; Table S1: Physicochemical properties of obtained PAL.
