# Supplementary material for: The Effect of Liquids Activated by Plasma Generated with a Microwave Plasmatron and High-Frequency Glow Discharge on Cotton Plant Development
Source: Plants (Basel). 2025 Jan 21;14(3):304. doi: 10.3390/plants14030304 (PMC11819904; doi:10.3390/plants14030304)
Supplement: Supplementary file 1 [file plants-14-00304-s001.zip › plants-3244561-supplementary.pdf]

Table S1. Physicochemical properties of obtained PAL.

Type of PAL	PAW	PAKNO$_3$
NO_2^-, μM	200 ± 20	1320 ± 95
NO_3^-, μM	100 ± 10	190 ± 28
H_2O_2, μM	150 ± 20	1.1 ± 0.4
Conductivity, S cm^{-1}	0.06 ± 0.002	0,035 ± 0.002
pH	5.6 ± 0.2	10.8 ± 0.2

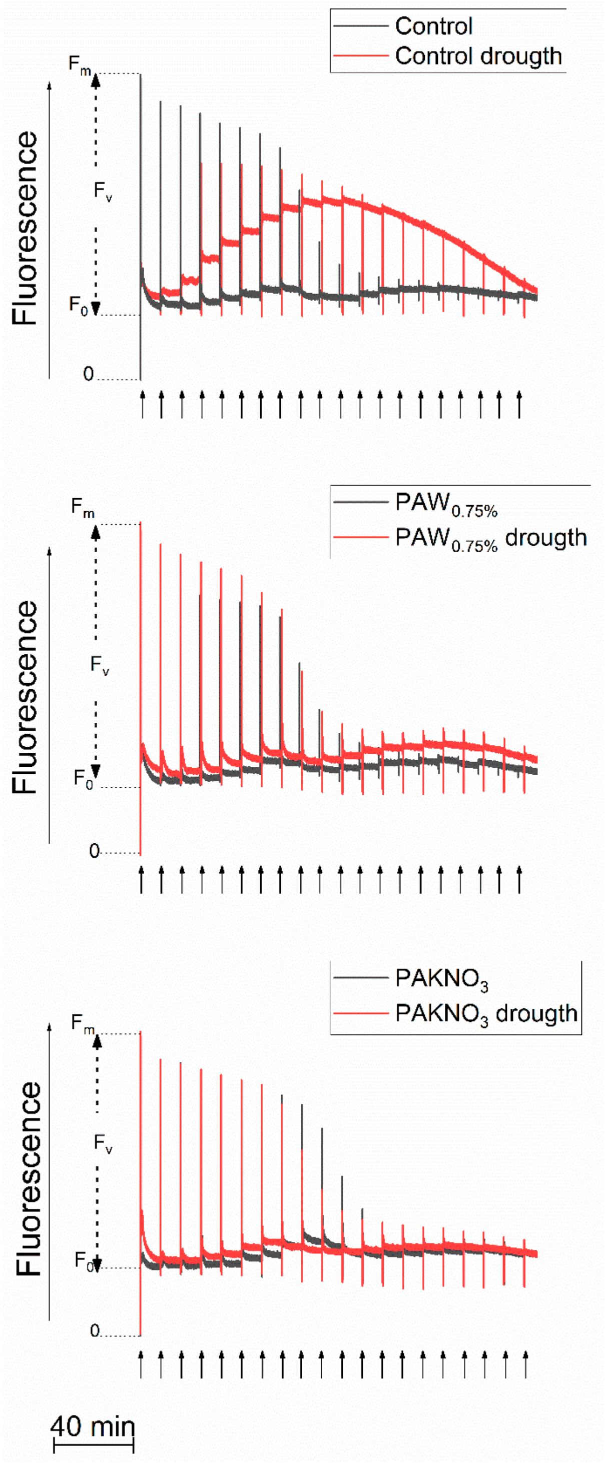

Figure S1. Representative kinetics of photoinduced changes of chlorophyll fluorescence yield (F_v) related to photoreduction of the primary electron acceptor Q_A. F_0 – the level of fluorescence induced by the measuring light; F_m – the level of fluorescence induced by a single saturating pulse in dark-adapted (60

min) samples, ↑ – a single 200 ms saturating flash (λ = 625 nm, 12,000 μmol photon s^{-1} m^{-2}), then shut down actinic light (AL), then inclusion far red light (FRL), then shut down (FRL) and inclusion AL. Plant adaptation to each intensity of AL (λ = 625 nm) was 10 minutes. Measurements were carried out at 27 °C, 40% humidity and CO_2 concentration of 400 ppm and repeated 3 times.
